# Supplementary figures and images for: High-Resolution Genetic Map and QTL Analysis of Growth-Related Traits of Hevea brasiliensis Cultivated Under Suboptimal Temperature and Humidity Conditions
Source: Front Plant Sci. 2018 Aug 24;9:1255. doi: 10.3389/fpls.2018.01255 (PMC6117502; doi:10.3389/fpls.2018.01255)

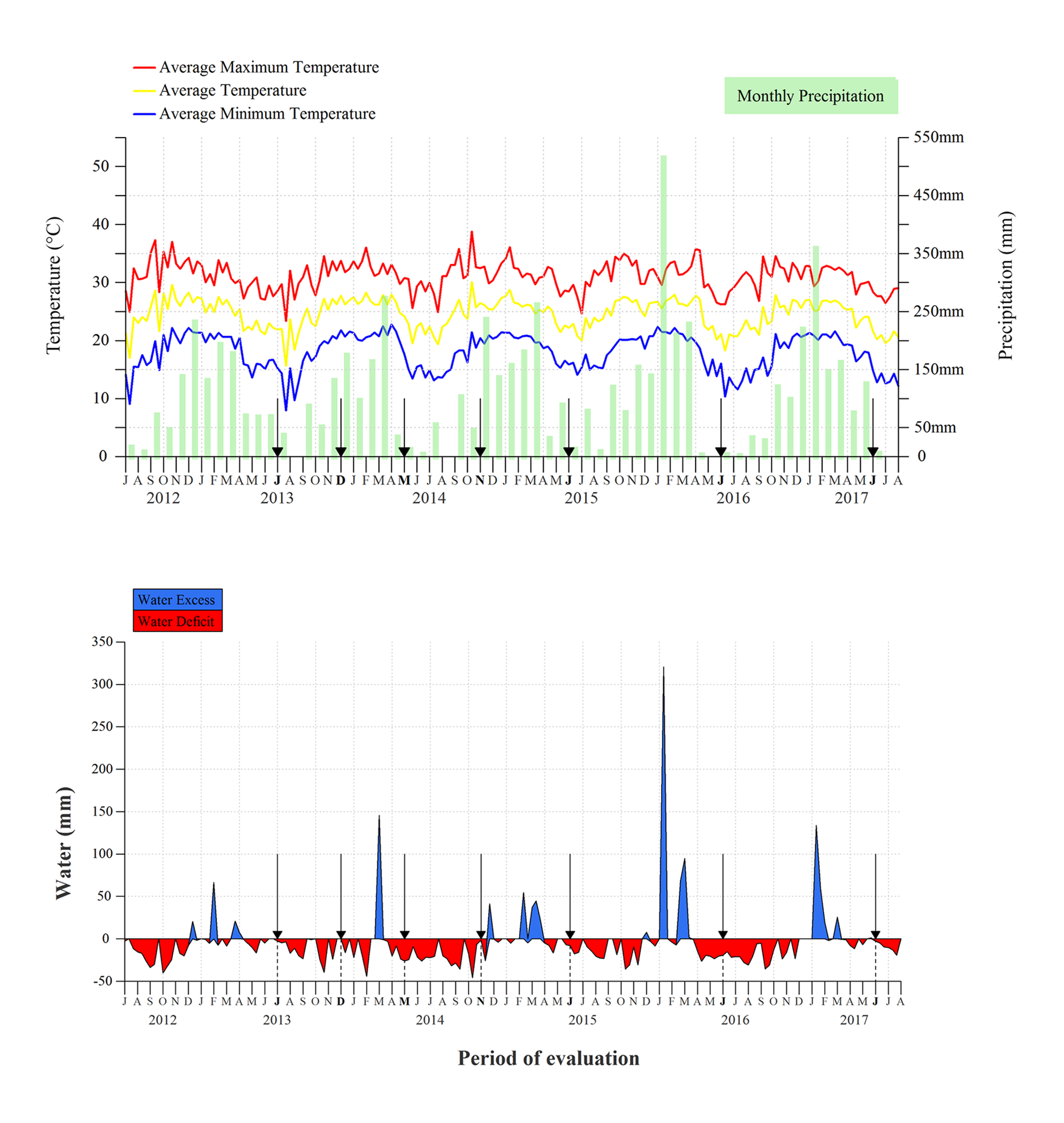

Supplement: FIGURE S1 — Temperature, precipitation and climatological water balance between July 2012 and August 2017 in Votuporanga, SP, Brazil. (A) Average maximum temperature, average temperature, average minimum temperature and monthly precipitation. (B) Evaluation of water excess and deficit periods. Black arrows indicate phenotypic measurements. [file Image_1.TIF]

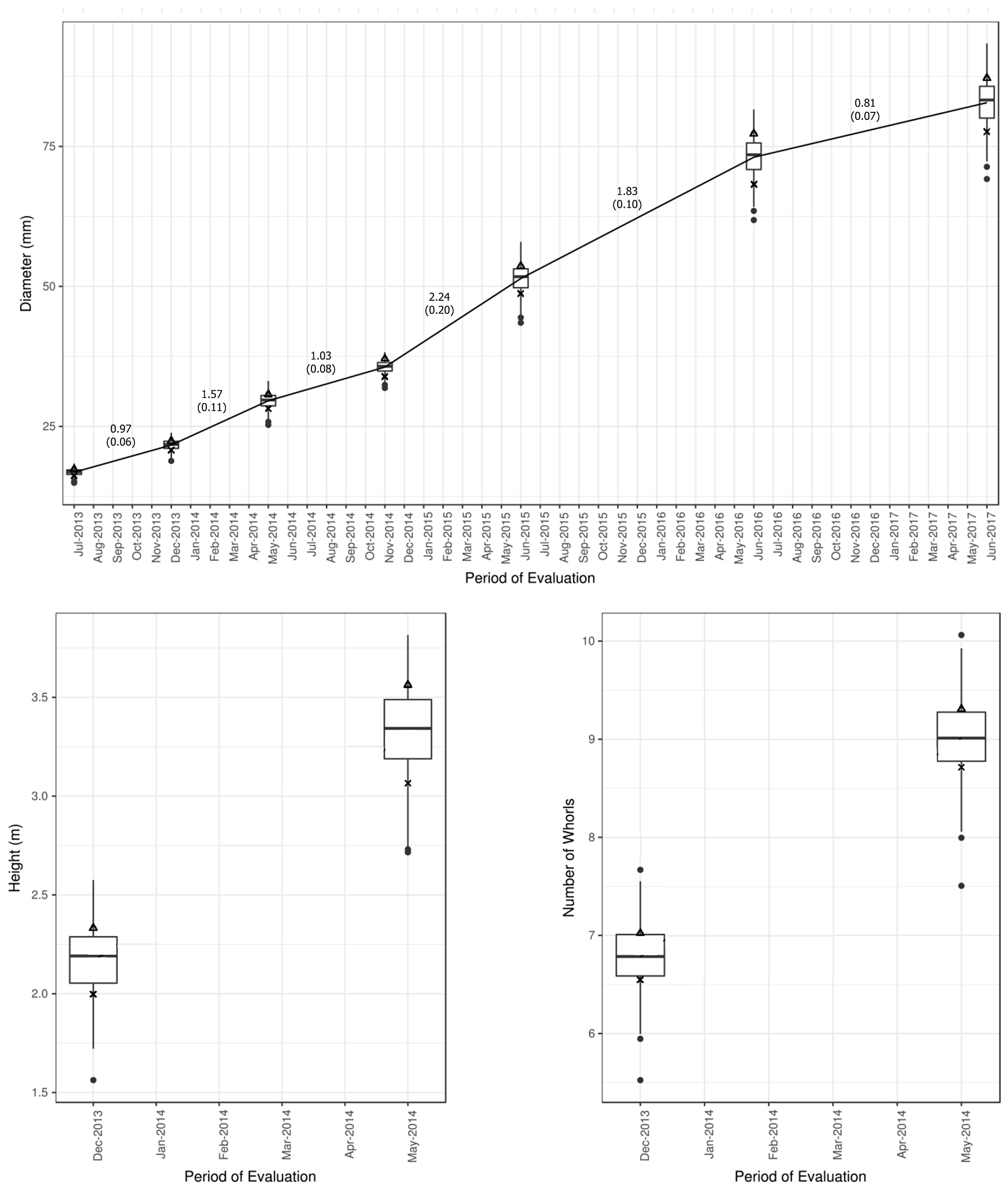

Supplement: FIGURE S2 — Boxplot of the genotypic predicted values for diameter (mm), height (m), and the number of whorls of the F1 population along the months of growth. The genotypic predicted value of the F1 parents RRIM 701 (cross) and GT1 (triangle) are also presented. Growth rates in millimeters per month (mean and standard deviation) are shown between each boxplot. [file Image_2.TIF]
